# Hemodynamic effects of perfusion level of peripheral ECMO on cardiovascular system

**DOI:** 10.1186/s12938-018-0493-5

**Published:** 2018-05-09

**Authors:** Kaiyun Gu, Zhe Zhang, Bin Gao, Yu Chang, Feng Wan

**Affiliations:** 1 Peking University Third Hospital, 49 North Garden Rd., Haidian District, Beijing, 100191 China; 2 Peking University Health Science Center, Xueyuan Rd, Haidian District, Beijing, 100083 China; 3 College of Life Science & Bio-Engineering, Beijing University of Technology, Beijing, 100124 China

**Keywords:** Hemodynamics, Peripheral ECMO, Blood assist index, Oscillatory shear index

## Abstract

**Background:**

Peripheral ECMO is an effective cardiopulmonary support in clinical. The perfusion level could directly influence the performances and complications. However, there are few studies on the effects of the perfusion level on hemodynamics of peripheral ECMO.

**Methods:**

The geometric model of cardiovascular system with peripheral ECMO was established. The blood assist index was used to classify the perfusion level of the ECMO. The flow pattern from the aorta to the femoral artery and their branches, blood flow rate from aorta to brain and limbs, flow interface, harmonic index of blood flow, wall shear stress and oscillatory shear index were chosen to evaluate the hemodynamic effects of peripheral ECMO.

**Results:**

The results demonstrated that the flow rate of aorta outlets increased and perfusion condition had been improved. And the average flow to the upper limbs and brain has a positive correlation with BAI (r = 0.037, p < 0.05), while there is a negative correlation with lower limbs (r = − 0.054, p < 0.05). The HI has negative correlation with BAI (p < 0.05, r < 0). The blood interface is further from the heart with the BAI decrease. And the average WSS has negative correlation with BAI (p < 0.05, r = − 0.983) at the bifurcation of femoral aorta and has positive correlation with BAI (p < 0.05, r = 0.99) at the inner aorta. The OSI under different BAI is higher (reaching 0.4) at the inner wall of the aortic arch, the descending aorta and the femoral access.

**Conclusions:**

The pathogenesis of peripheral ECMO with different perfusion levels varies; its further research will be thorough and extensive.

## Background

Veno-arterial extra corporeal membrane oxygenation (VA ECMO) is a common treatment for respiratory failure and heart failure clinically [1–4]. VA-ECMO drains the blood from the venous vessels and then returns the processed blood to the arterial vessels and this has an advantage of pulmonary as well as cardiac support. Peripheral ECMO is one of the VA ECMO mode which usually has an arterial cannula placed into the right femoral artery for infusion and a venous cannula placed in the right common femoral vein for extraction [5–8]. Peripheral ECMO is usually used in cardiogenic shock and cardiac arrest and leaves small surgical wound.

Lower extremity ischemia, amputation and vascular complication are common complications of ECMO treatment [9]. High blood flow velocity from the ECMO cannula flow may result in high wall shear stress of local vascular and high blood pressure which may result in vascular complication [10]. The retrograde ECMO flow also increases the left ventricular afterload [11], even restrict the opening of the aortic valve [12, 13]. The flow interface caused by ECMO jet flow and cardiac jet flow may also lead to severe flow conditions resulting in platelet activation or hemolysis. Coronary arteries, cerebral blood vessels and upper limbs may be also under threat of hypoxaemia for proximal branches of the aorta receive predominantly deoxygenated blood ejected from the heart [14]. Limb ischemic is a common complication with peripheral ECMO and more than half of our patients developed it [15–17]. Peripheral ECMO may not well supply the coronary arteries and proximal aorta arch branches and these vessels may be filled with poorly oxygenated blood by the heart if the patient has respiratory failure. The non-pulsatile blood flow of ECMO is another risk for it alters the pulsatile flow patterns and cerebral autoregulation [18]. Thus, the possibility of complications of ECMO have relationship with the perfusion condition of ECMO. In clinical, the perfusion amount is based on doctors’ own experience, the hemodynamic performances under different perfusion level of ECMO have been less studied.

Computational fluid method has been widely used in hemodynamic study of cardiovascular system and related assist devices. The resulting hemodynamic vectors, wall shear stress, pressure gradients and other factors were obtained and analyzed to investigate the effects of ECMO on the blood and vascular. Avrahami et al. [19, 20] analyzed hemodynamic characteristics of aorta cannula and the risk to develop cerebral embolism and hemolysis. Menon et al. [21] also study the jet wake of aortic cannula and related disease.

This article used computational fluid dynamic method [22–25] to clarify the hemodynamic performance of peripheral ECMO under different perfusion level, and to further guide its empiric therapy scientifically. Specially, the hemodynamic analyse was compared under different blood perfusion conditions. That is, a blood assist index (BAI) was defined to represent the ratio of ECMO energy to total energy. Four numerical simulations were performed under different BAI (80, 60, 40, 0%) and the results were compared.

## Methods

### Define of the blood assist index (BAI)

In order to evaluate support level of ECMO, the blood assist index (BAI) was defined to represent the ratio of ECMO energy to total energy, denoted as Eq. 1.

1 $$BAI = \frac{1}{T}\int_{0}^{{T_{c} }} {\left( {\frac{{F_{E} (t)}}{{F_{E} (t) + F_{C} (t)}}} \right)dt,}$$

where $F_{E} (t)$ is the waveform of ECMO cannula outlet blood flow, $F_{C} (t)$ is the waveform of cardiac output blood flow, $T_{c}$ represents the cardiac cycle. The unit of BAI is %. It is seen that when the BAI equals to 1, the ECMO is fully assisted and the cardiac output is zero. When the BAI is lower than 1, the ECMO is partial assisted. When the BAI is zero, only the native heart supplies the body. In this study, to meet the physical requirement, the total blood perfusion is set about 5 L/min. for different perfusion conditions were assumed as BAI of 80, 60, 40 and 0%. That is, the contribution of the ECMO is weaker with the lower of BAI.

### Computational fluid dynamic method

**Fig. 1:**
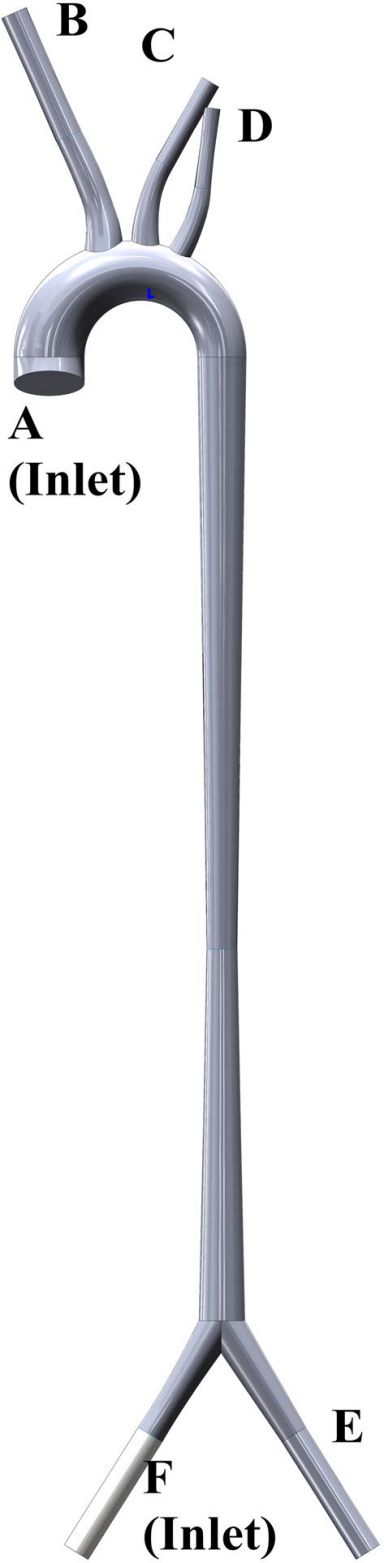
The ideal three-dimensional geometry of peripheral ECMO cannulation and related blood vessels

In order to investigate hemodynamic performance of the peripheral ECMO under different perfusion conditions, several numerical simulations were conducted. The three-dimensional geometry of peripheral ECMO cannulation was created as shown in Fig. 1. The vessel size refers to literatures [26] as Table 1.

**Table 1:** The vessel sizes of the geometry

	Location	Diameter (mm)
A	Inlet of aorta	28
B	Outlet of innominate artery	7
C	Outlet of left common carotid artery	7
D	Outlet of left subclavian artery	7
E	Outlet of left femoral artery	10.3
F	Diaphragm	11.3

To determine the number of meshes of the model, having a good quality and little influence on the results of CFD simulation, a gird independent test was conducted. Five models with different numbers of element, from 1.4 to 8.1 millions, were established. The steady flow numerical studies, which include an inlet boundary condition of constant velocity flow rate, and 70 mmHg pressure for the outlets, was used to the analysis. The pressure at the inlet plane of aorta and the average wall shear stress of the whole vessel are chosen as the indicators. The relative error of them has been used to evaluate the accuracy of simulation. The test results were listed in Table 2. It is shown that both relative errors of the pressure and wall shear stress decreased to less than 1%, when the numbers of elements is more than 2,358,058. Hence the mesh containing 2,358,058 elements has been used in this work.

**Table 2:** The results of analysis of grid independence

Number of elements	Pressure at the inlet of aorta (Pa)	Relative error of pressure (%)	Average wall shear stress (Pa)	Relative error of WSS (%)
1,406,843	9430.16	–	0.431802	–
2,358,058	9442.28	1.68	0.424562	0.43
4,758,418	9442.36	0.54	0.426851	0.02
6,663,614	9442.24	0.10	0.426389	0.0083
8,156,540	9442.56	0.18	0.427151	0.0098

There are four simulations cases conducted in this work. Cases 1–4 is the simulation of the peripheral ECMO under BAI of 80, 60, 40 and 0%.

**Fig. 2:**
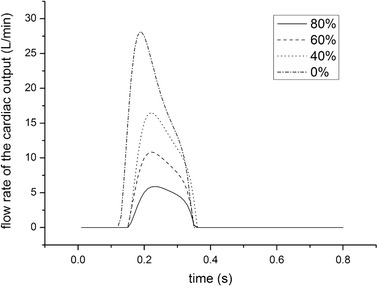
The inlet flow rate waveforms of the aorta of different BAI

The boundary conditions were set as pulsatile velocity inlet and fully developed constant pressure outlet. The inlet flow rate waveforms of the aorta were assumed to be transient which was obtained from the lumped parameter model (LMP) [27–29] as shown in Fig. 2. The period of the pulsatile flow is set as 0.8 s, which is equal to the cardiac period. The sum of the average flow rate of ECMO cannula and cardiac output keeps constant, thus the total blood perfusion is 5 L/min meeting the physical requirement. The outlet pressure was set as 70 mmHg for all vessel outlets.

In all simulation cases, the blood was assumed as a homogeneous, incompressible, and Newtonian fluid flow for reducing the cost of computation. And the density and viscosity of blood is set as 1050 kg/m^3^ and 0.0035 kg/m s, respectively [25]. Therefore, blood flow was modeled using incompressible Navier–Stokes equations in moving boundaries.

### Definition of indicators of hemodynamic performance

To assess the perfusion condition main vessels under ECMO, blood flow rate ratios (R) of arterial bifurcations was defined.

*R*_*up*_ is the ratio of the upper limb and brain blood supply to total blood supply. *R*_*down*_ is the ratio of the lower limb blood supply to total blood supply. They were defined as Eqs. 2 and 3.

2 $$R_{up} = \frac{{Q_{innominateartery} + Q_{leftcommoncarotidartery} + Q_{leftsubclavianartery} }}{{Q_{aorta} }}$$

3 $$R_{down} = \frac{{Q_{leftfemoralartery} + Q_{rightfemoralartery} }}{{Q_{aorta} }}.$$

To evaluate the pulsatility of the flow rate, the harmonic index (HI) was proposed. HI is a measure of the relative contribution of non-static intensity to the overall signal intensity, and this parameter ranges from zero (in the case of a steady nonzero flow rate signal) to one (in the case of a purely oscillatory signal with a time average of zero) [19]. The harmonic index (HI) is defined as Eq. 4:

4 $$HI = \frac{{\sum\nolimits_{n = 1}^{ + \infty } {T[nw_{0} ]} }}{{\sum\nolimits_{n = 0}^{ + \infty } {T[nw_{0} ]} }},$$

where $T[nw_{0} ]$ is the magnitude of the transformed flow rate signal.

To clarify the flow oscillation during cardiac cycle and quantify the change in direction and magnitude of the WSS, the oscillatory shear index (OSI) was calculated as Eq. 5 [20]:

5 $$OSI = \frac{1}{2}\left( {1 - \frac{{\left| {\int_{0}^{T} {\tau_{w} dt} } \right|}}{{\int_{0}^{T} {\left| {\tau_{w} } \right|dt} }}} \right)$$

where $\tau_{w}$ is wall shear stress, *T* is one cardiac cycle. The OSI value can vary from 0 to 0.5, where 0 describes a total unidirectional WSS and the latter a purely unsteady, oscillatory shear flow with a net amount of zero WSS. Areas of high OSI are predisposed to endothelial dysfunction and thermogenesis [21, 22].

## Results

**Fig. 3:**
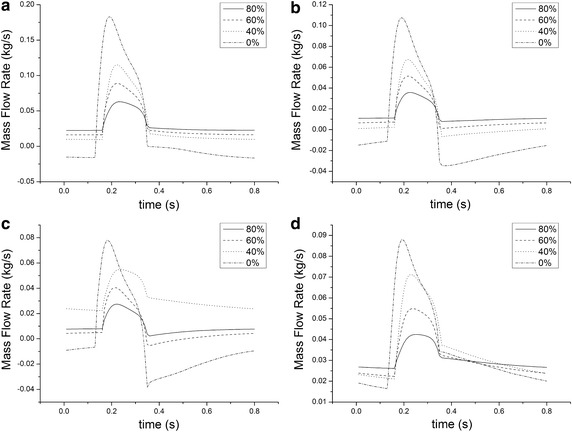
The flow rate of all the outlets under different BAI. **a** The flow rate of innominate artery, **b** the flow rate of left common carotid artery, **c** the flow rate of left subclavian artery, **d** the flow rate of left femoral artery

Figure 3 shows the flow rate of all the outlets of the cases. Figure 3 is the mass flow rate of the peripheral ECMO under different BAI. The flow rate of the outlets is changing with time and the changing trend is related with the inlet flow of the aorta. There is descend value around 0.4 s due to the end of cardiac ejection. The peak values of flow rate of all the outlets increase with the decrease of BAI. The peak value of innominate artery (IA) is much higher than other outlets. Without ECMO (0% BAI), there exists backflow moment of IA, left common carotid artery (LCCA) and left subclavian artery (LSA). While for peripheral ECMO, along with the increase of BAI, the backflow situation gets better with the increase of BAI, and under 80% BAI, there is no backflow moment of all the outlets. With ECMO assistance, the backflow condition had been improved.

**Fig. 4:**
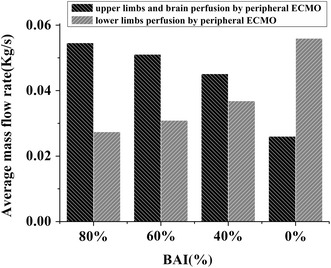
The average mass flow rate under different BAI

Figure 4 is the average mass flow rate under different BAI. The blood flow of the IA, LCCA and LSA supply the upper limbs and brain. The blood flowing into the left femoral artery (LFA) and right femoral artery (RFA) perfuse the lower limbs. And the average flow to the upper limbs and brain has a positive correlation with BAI of ECMO (r = 0.037, p < 0.05), while there is a negative correlation with lower limbs (r = − 0.054, p < 0.05).

**Fig. 5:**
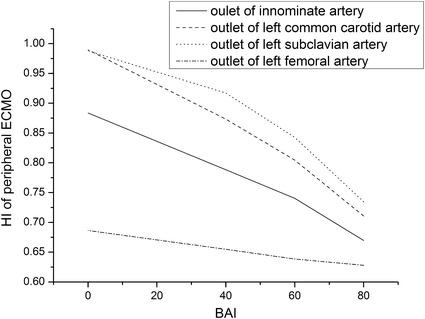
The HI of all the flow rate waves under different BAI

Figure 5 is the HI of all the flow rate waves under different BAI. The HI decreases with the increase of BAI.

**Fig. 6:**
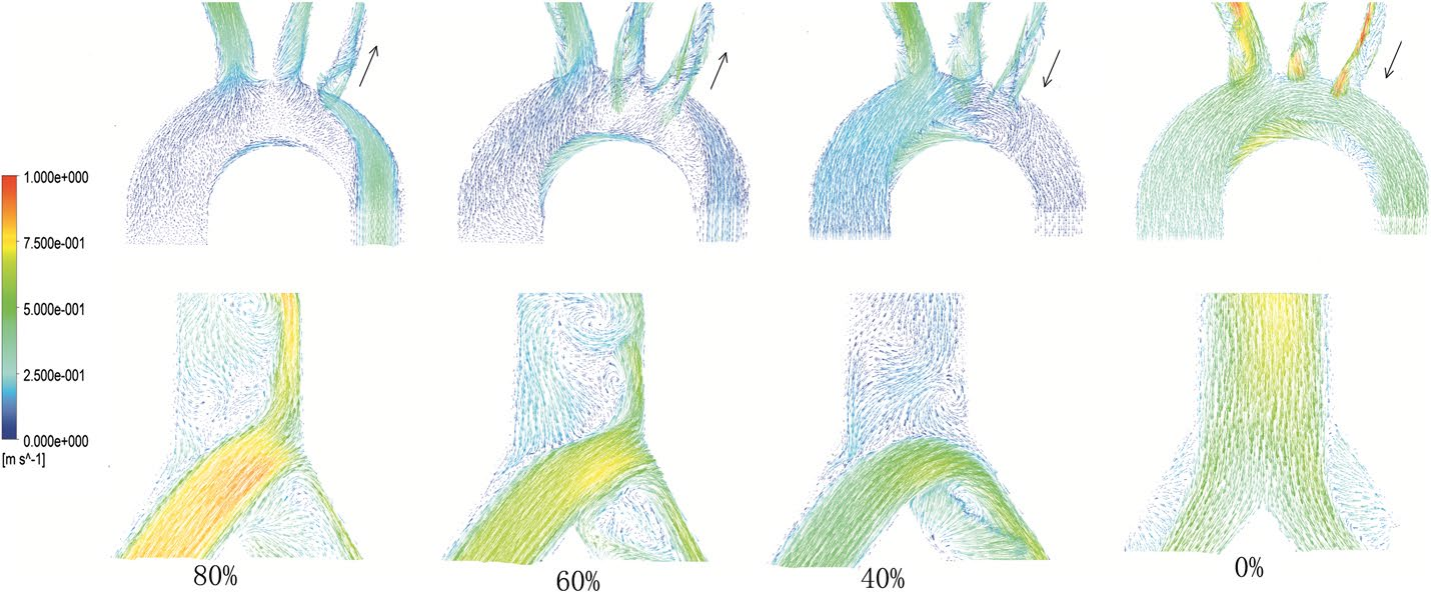
The velocity vector of aorta arch and femoral bifurcation at peak moment under different BAI

**Fig. 7:**
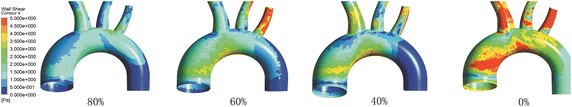
The wall shear stress (WSS) contours of aortic arch at feature moments under different BAI

Figure 6 is the velocity vector of aorta arch and femoral bifurcation at peak moment. With the BAI increases, the energy of ECMO becomes weaker and the energy of local heart becomes stronger. There is backflow at the inside of the aorta bent. With the BAI gets lower, the vortex in the aorta arch becomes stronger. At the bifurcation of femoral, there exists vortex near the jet flow of ECMO. Seen from Fig. 7, the blood interface changes with the BAI, and with the BAI decrease, the location is further from the heart, the backflow inside the aorta bent is stronger.

**Fig. 8:**
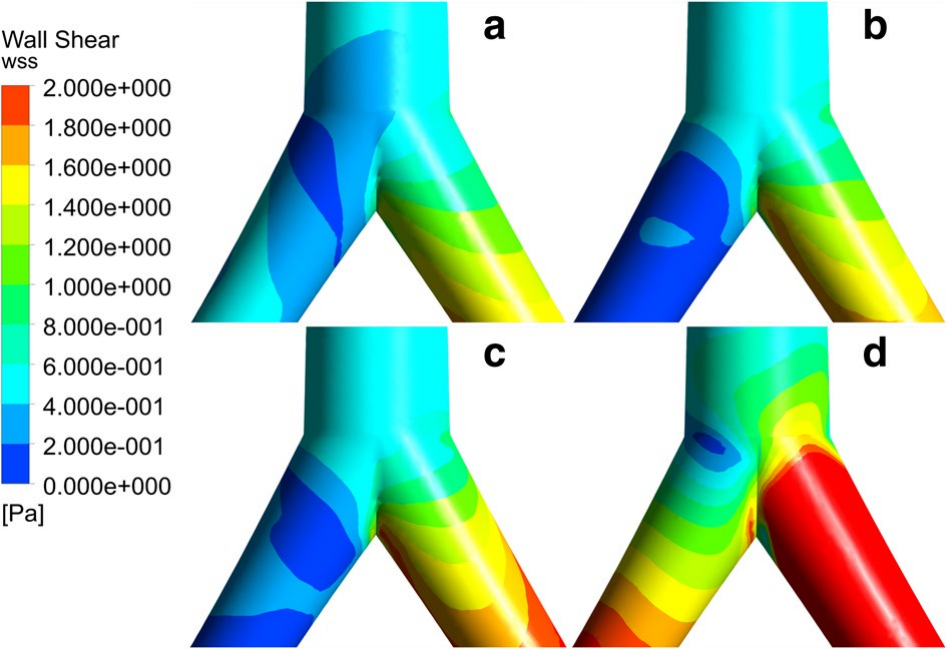
The wall shear stress (WSS) contours at the bifurcation of femoral artery under different BAI. **a** The WSS under 0% BAI, **b** is the WSS under 40% BAI, **c** the WSS under 60% BAI, **d** the WSS under 80% BAI

Figures 7 and 8 illustrate the wall shear stress (WSS) contours at feature moments under different BAI. The inside aorta bent (Fig. 7) and side of femoral (Fig. 8) are high WSS region of femoral ECMO. The WSS distribution also changes with BAI. For aorta arch, the WSS of the inner aorta arch and arterial bifurcation increase with the BAI decreases. The WSS of femoral bifurcation decrease with the BAI decreases and it is higher than aorta arch. Table 3 is the average WSS of the both regions.

**Table 3:** The average WSS under different BAI (pa)

BAI (%)	Aortic arch	Femoral artery
80	0.289	1.141
60	0.395	0.909
40	0.487	0.7779
0	0.590	0.176

**Fig. 9:**
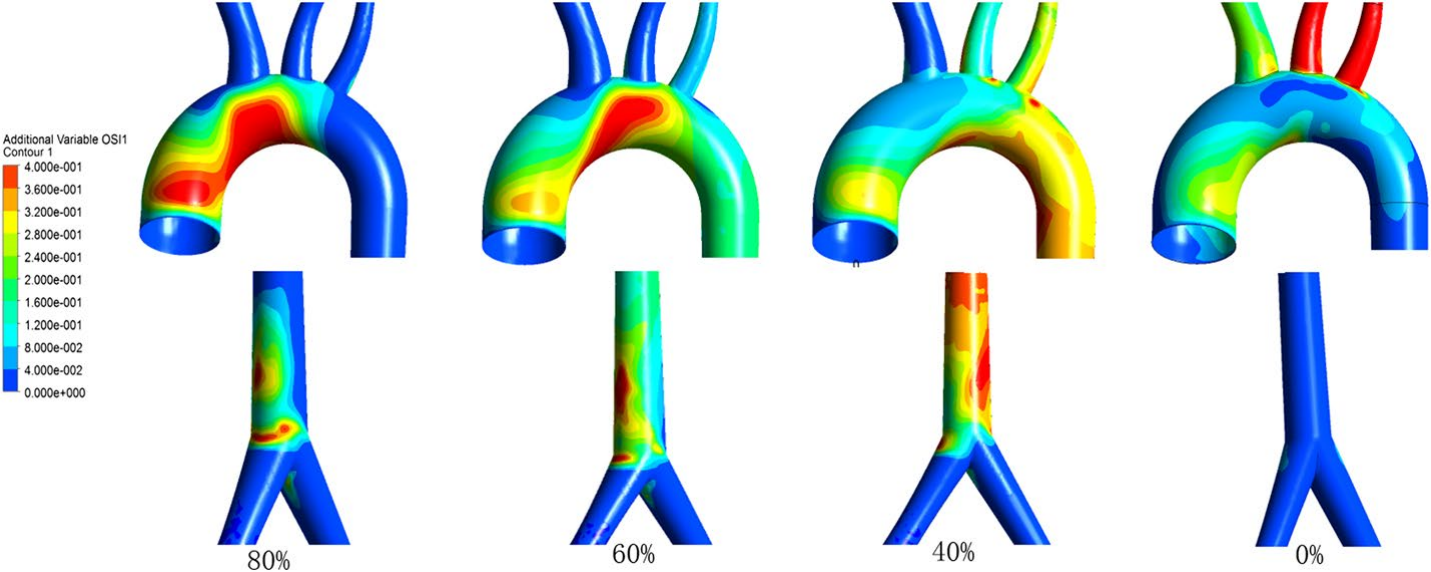
The OSI under different BAI

Figure 9 is the OSI under different BAI. With the BAI decrease, for the inner wall of the aortic arch, the OSI decrease, and for the descending aorta, the OSI increase, and for the femoral access, the OSI increase.

## Discussion

VA ECMO is an effective treatment for severe cardiopulmonary failure, but the complications of ECMO threaten the life of patients. Except bleeding and infection which are mainly caused by the operation, other complications, such as ischemia, hypoxaemia, hyperperfusion and vascular complication, have relationship with the hemodynamic flow field. This article focuses on the hemodynamic difference of the peripheral ECMO under different BAI. CFD was used to solve these problems obtaining the hemodynamic results of the two modes and BAI was defined to represent the perfusion condition of ECMO. Hemodynamic factors different BAI were compared. Although the effect of ECMO on cardiovascular system has attracted many interesting, the relationship between support level of ECMO, blood distribution and hemodynamic states are not clear. This is the first paper revealing these relationships by using numerical method.

Limb ischemia is a common complication of ECMO and it may lead to limb loss even death in more serious cases. Slottosch et al. [30] reported that 20.8% of the patients undergoing peripheral ECMO required treatment of lower limb ischemia. Cheng et al. [31] stated that for peripheral ECMO 16.9% of patients meet lower extremity ischemia and 4.7% of patients have lower extremity amputation. Distal perfusion catheters were proposed to improve this situation [32], but there is still a 3.2% rate of limb ischemia even though distal perfusion catheters were implanted [33]. Cerebral blood vessels and upper limbs are also under the threat of hypoxaemia undergoing peripheral ECMO [34] for proximal branches of the aorta receive predominantly deoxygenated blood ejected from the left heart and this situation persists as long as the return cannula is placed centrally [35]. Our results suggest that blood perfusion to limbs is correlation with BAI (p < 0.05), and for lower limbs there is negative correlation between BAI and flow rate while for upper limbs and brain it is positive correlated (Fig. 4). These results are consistent with clinical events listed above. The results of this study show that the support level could directly affect the distribution of blood between upper limbs and lower limbs. That means, the output of ECMO should be regulated in response to the changes in both perfusion requirements and cardiac function to achieve an optimal clinical outcome.

For peripheral ECMO, there exists blood interface due to the different direction of blood supply from heart and peripheral ECMO. After cardiac injection, the perfusion from the heart becomes weaker, retrograde flow from peripheral ECMO reaches aorta arch meeting the antegrade flow from heart forming the interface. The peripheral ECMO mainly supply the blood to the bifurcation of the arch. The flow interface caused by ECMO jet flow and cardiac output flow is another factor that influences the perfusion under peripheral ECMO. Seen from Fig. 6, the closer the location of the interface is to the heart, the higher the BAI and the interface reaches the branches of aorta when BAI exceeded 40%. It may be used to explain the growth of the flow rate to upper limbs and brain under peripheral ECMO slows down when the BAI is greater than 40%. In addition, when BAI is set as 40%, a vortex is found under the inlet of left subclavian artery, which has generated regurgitation flow. Hence, the support level of peripheral ECMO should be regulated to avoid the vortex closing to the inlet of upper vessels.

The human heart produces pulse by contraction, stroke volume ejection, and then relaxation, with one-way valves. Given this mechanism, a pulsatile circulation is obligatory [36]. The non-pulsatile blood flow from the ECMO may generate negative effects on heart and aorta. Short et al. [37] have shown that VA ECMO alters pulsatile blood flow patterns and cerebral autoregulation in animal models of VA ECMO for the effect on endothelial reactivity. HI is an index evaluating the pulsatility of the flow rate. The higher the HI, the stronger the pulsatility. HI has negative correlation with BAI (p < 0.05, r < 0).

Wall shear stress has relationship with vascular remodeling, which is an implication on atherosclerosis, coronary stents, VAD and ECMO [38]. Mean and maximum values of WSS together with WSS amplitude are major determinants of endothelial pathology [39, 40] and intimal disease [41]. Adel et al. [42] indicated that arterial-level shear stress (> 1.5 pa) induces endothelial quiescence and an atheroprotective gene expression profile, while low shear stress (< 0.4 pa), which is prevalent at atherosclerosis-prone sites, stimulates an atherogenic phenotype. Seen from Table 3, our results shows that under the average WSS at region 2 during one cardiac cycle under 0% of BAI was all higher than 1.5 pa. That is without ECMO, the WSS was in the safe range for endothelial cell. For peripheral ECMO, the average WSS of region 1 is lower than that without ECMO while the average WSS of region 2 is much higher. And the average WSS has negative correlation with BAI (p < 0.05, r < 0). For peripheral ECMO, the OSI of the aorta arch and access of femoral artery is higher than normal state. And it shows that the areas with high values of OSI are usually located in the regions where wall shear stress is low. It is constant with other studies [43]. With the BAI decrease, the OSI in the inner aorta decrease while the WSS increase. The inlet flow of peripheral ECMO canula causes high OSI compared with 0% of BAI. Areas of high OSI are predisposed to endothelial dysfunction and atherogenesis.

Flow-imaging techniques such as phase contrast magnetic resonance imaging is performed to produce flow fields of blood. The state of change in swirling blood flow within cardiac chambers, flow information by overlaying velocity fields, and to quantify it for clinical analysis was studied by Wong [44, 45]. It was establish a framework to produce flow information and set of reference data to compare with unusual flow patterns due to cardiac abnormalities. In addition, Du propose a regression segmentation framework (Bi-DBN) to automatically segment bi-ventricle by establishing a boundary regression model that implicates the nonlinear mapping relationship between cardiac MR images and desired object boundaries [46]. Zhang propose meshfree particle computational method for cardiac image analysis with the energy minimization formulations to solve the fundamental problem about the optimal mathematical description in cardiac image analysis on a digital computer [47]. In this paper, computational fluid method was used to research the hemodynamic effects of perfusion level of peripheral ECMO. In the future, we will try to use bi-ventricle segmentation and meshfree particle computational method in my research. Combine the flow-imaging techniques and computational fluid method to measurement and analysis the flow will be used in this field.

The idealistic geometric can capture most of important characteristic of the problem. For computational fluid dynamic, idealistic geometric is also simple and easier to implement [48, 49]. In this work, the ideal 3-dimensional geometry model is established, consisting the ascending aorta, the innominate artery (IA), left common carotid artery (LCCA), left subclavian artery (LSA), left femoral artery (LFA), right femoral artery (RFA). Hence, the accuracy of results may be limited by this choice. However, this work is focused on the common relationship between perfusion level and cardiovascular system, rather than the hemodynamic effect on specific patients. Hence, these results also could reveal the mechanisms on hemodynamic status under peripheral ECMO support in some degree. In the future, the MRI data will be used to obtain motion of coronary wall and the fluid structure interaction method (FSI) will be used to study the hemodynamic states of coronary artery.

Cardiovascular disease is still the leading cause of death over the world. There are significant challenges including the real-time monitoring of physiological states, imaging technologies and personalized predication [50]. According to literatures, the hemodynamic states have strong effects on function and structure of cardiovascular system, such as the auto-regulation system, the function of aortic valve and the brain perfusion. These effects are very important for the long-term prognosis outcome of patients. Then, computer modeling supplies an opportunity for parameters of hemodynamic states to provide a quantitative assessment [51]. This work is mainly focused on the hemodynamic effect caused by different support level, hence the physiological effects was not studied in this work. In the future, other study on the physiological effects of different support level of ECMO on cardiovascular structure and function will be conducted.

Moreover, the results is derived from numerical study (CFD). Although the CFD method could reveal many kinds of very useful information, the PIV method is still needed to be conducted to verify their accuracy and strengthen these results. Hence, the PIV study on the hemodynamic change under different support level of ECMO at aorta and femoral artery will be conducted.

## Conclusion

Peripheral ECMO is an effective cardiopulmonary support in clinical. In this paper, the effects of the perfusion level on hemodynamics of peripheral ECMO was studied.

The geometric model of cardiovascular system with peripheral ECMO was established. The blood assist index was used to classify the perfusion level of the ECMO. The flow pattern from the aorta to the femoral artery and their branches, blood flow rate from aorta to brain and limbs, flow interface, harmonic index of blood flow, wall shear stress and oscillatory shear index were chosen to evaluate the hemodynamic effects of peripheral ECMO. The results revealed the mechanisms on hemodynamic status under peripheral ECMO support in some degree, its further research will be thorough and extensive.
